# Supplementary material for: Changes in Expression of Complement Components in the Ovine Spleen during Early Pregnancy
Source: Animals (Basel). 2021 Nov 8;11(11):3183. doi: 10.3390/ani11113183 (PMC8614503; doi:10.3390/ani11113183)
Supplement: Supplementary file 1 [file animals-11-03183-s001.zip › Table S1 Primers.pdf]

Table S1 Primers used for RT-qPCR

Gene	Primer	Sequence	Size (bp)	Accession numbers
C1qA	Forward	CAGGAGAACGTGTACCAGAGCAAC	122	XM_012152629.2
	Reverse	CTCCGAGAGGACCTGATGGACAG		
C1r	Forward	CCCAGACTACCGCCAGGAAGAG	109	XM_012175492.2
	Reverse	TGGGAGGCAGATTGGCAGGAG		
C1s	Forward	CCTGGCAAGTCTTCTTCTCGAACC	130	XM_004006917.4
	Reverse	ACCACTGAGGAGGACCCAACATAC		
C2	Forward	CCACCAATCCCATCCAGCAGAAG	95	XM_012100922.4
	Reverse	GGCGTCCAGGAGCAGGTAGAG		
C3	Forward	CGCCACCAGCAGACTATAACGATC	105	XM_027969774.1
	Reverse	AGCAGCCTTGACCTCCACCTC		
C4a	Forward	TTCAGGACAGGTGGTGAGAGGATC	167	XM_027958803.1
	Reverse	GGAGGAGATGGAGGCGACAGAG		
C5b	Forward	GCTACGCTGGTGTTACTCTGGATC	157	XM_004003966.3
	Reverse	GCAGACATGACCTCGCCTATAAGC		
C9	Forward	GCCGCAACAGAGTGGTGGAAG	138	XM_004017026.3
	Reverse	TGCCATCCCTAACTCGGTCACAG		
GAPDH	Forward	GGGTCATCATCTCTGCACCT	176	NM_001190390.1
	Reverse	GGTCATAAGTCCCTCCACGA		
