# Supplementary material for: Changes in Expression of Complement Components in the Ovine Spleen during Early Pregnancy
Source: Animals (Basel). 2021 Nov 8;11(11):3183. doi: 10.3390/ani11113183 (PMC8614503; doi:10.3390/ani11113183)
Supplement: Supplementary file 1 [file animals-11-03183-s001.zip › Table S2 antibodies.pdf]

Table S2 Antibodies used for Western blot

Protein	Antibody
C1q	Mouse anti-C1q monoclonal antibody (Santa Cruz Biotechnology, Santa Cruz, CA, USA, sc-53544)
C1r	Mouse anti-C1r monoclonal antibody (Santa Cruz Biotechnology, sc-514105)
C1s	Mouse anti-C1s monoclonal antibody (Santa Cruz Biotechnology, sc-365273)
C2	Mouse anti-C2 monoclonal antibody (Santa Cruz Biotechnology, sc-373809)
C3	Mouse anti-C3 monoclonal antibody (Santa Cruz Biotechnology, sc-28294)
C4a	Mouse anti-C4a monoclonal antibody (Santa Cruz Biotechnology, sc-271181)
C5b	Mouse anti-C5b monoclonal antibody (Santa Cruz Biotechnology, sc-398247)
C9	Mouse anti-C9 monoclonal antibody (Santa Cruz Biotechnology, sc-390000)
GAPDH	Mouse anti-GAPDH antibody (Santa Cruz Biotechnology, sc-47724)
