# Supplementary figures and images for: Changes in Expression of Complement Components in the Ovine Spleen during Early Pregnancy
Source: Animals (Basel). 2021 Nov 8;11(11):3183. doi: 10.3390/ani11113183 (PMC8614503; doi:10.3390/ani11113183)

C1q

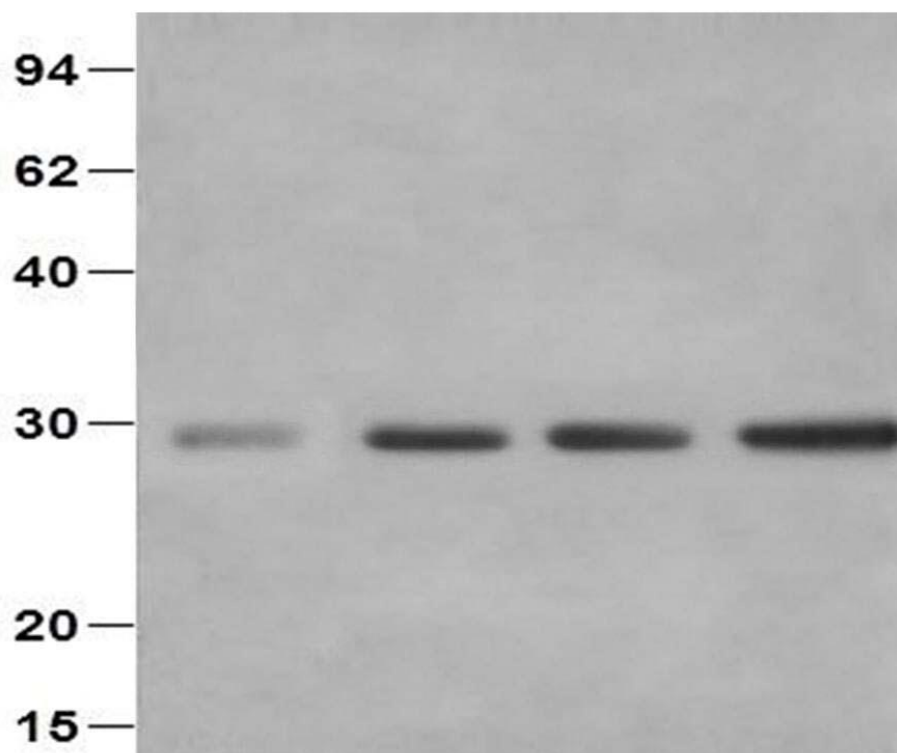

C1r

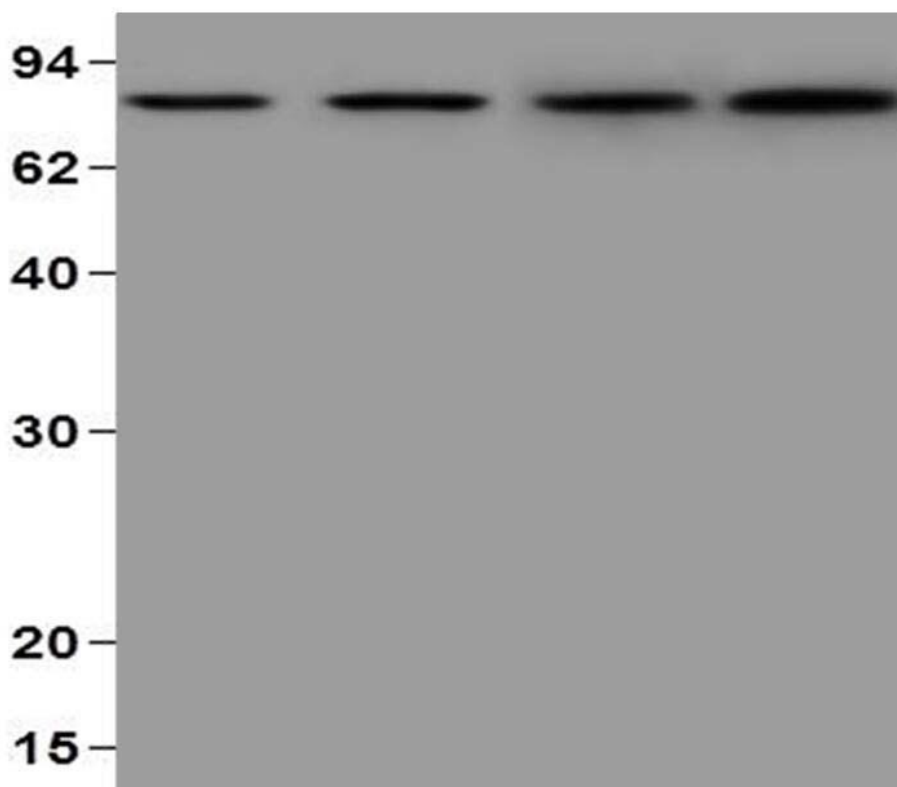

C1s

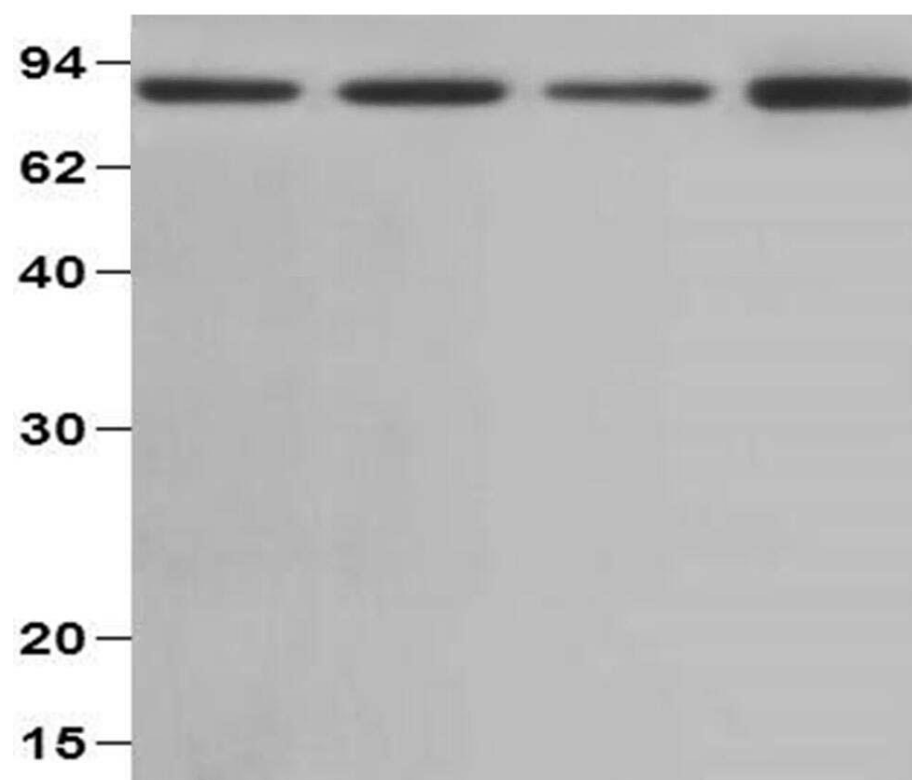

C2

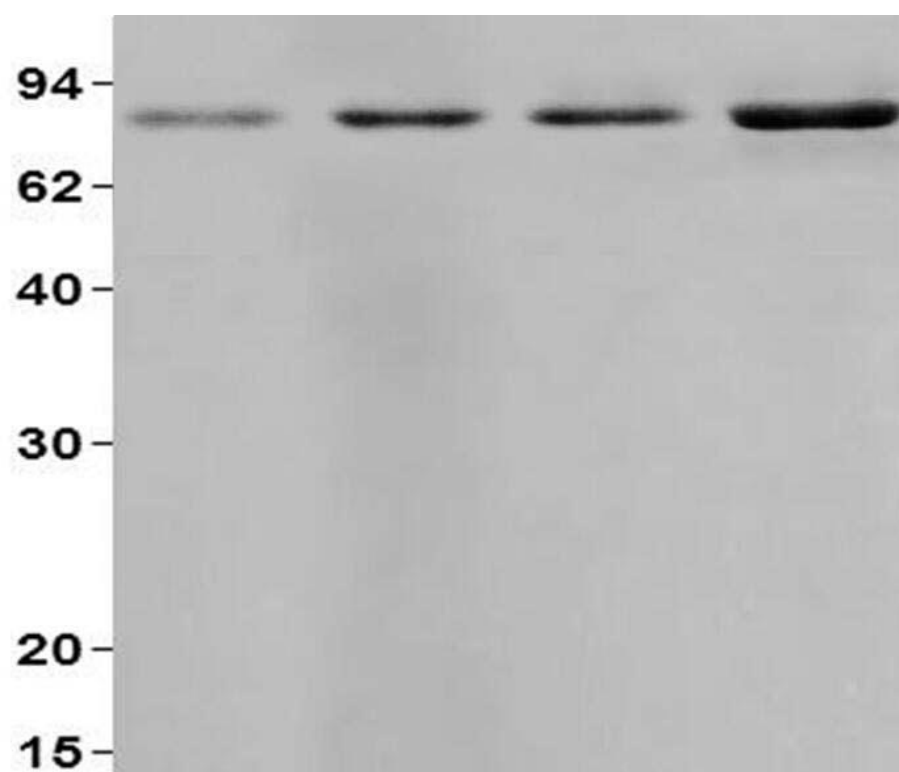

C3

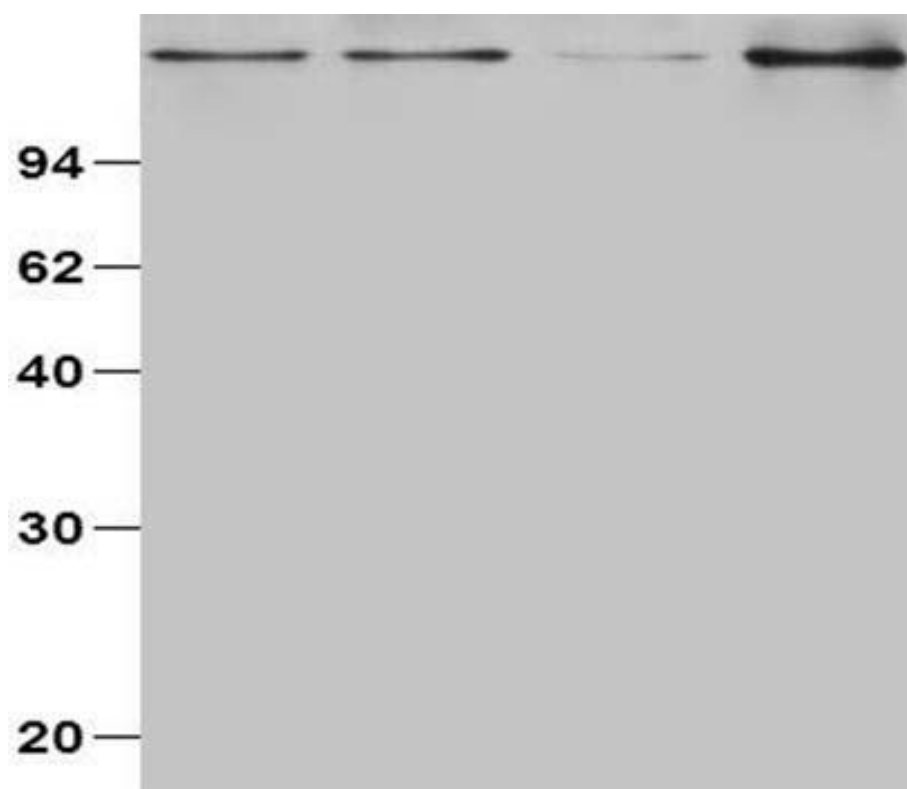

C4

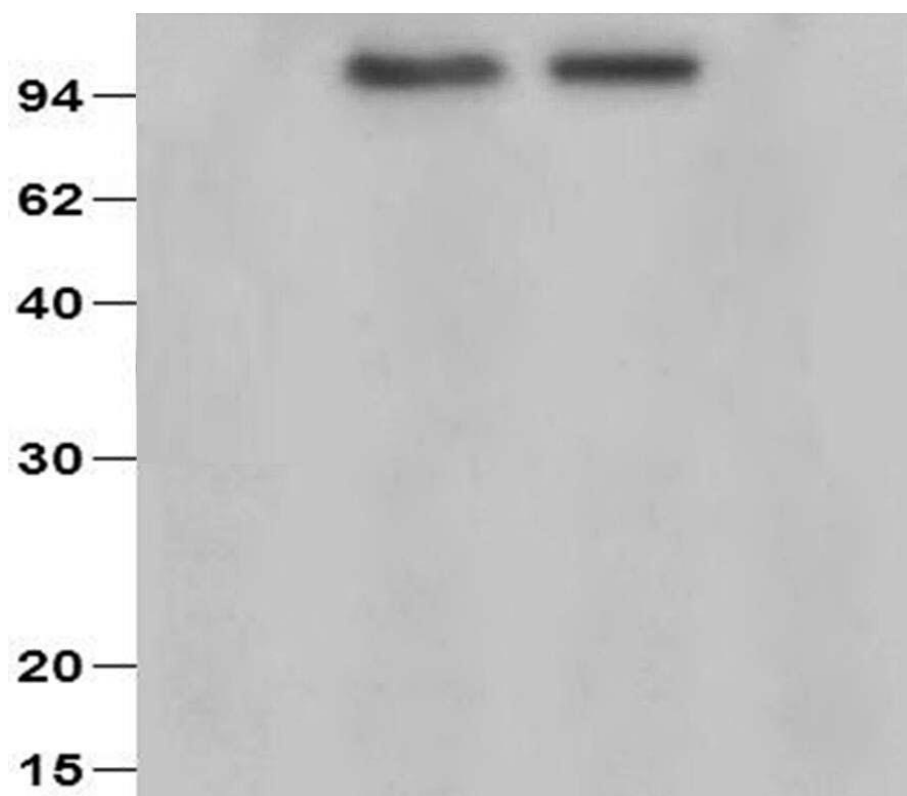

C5

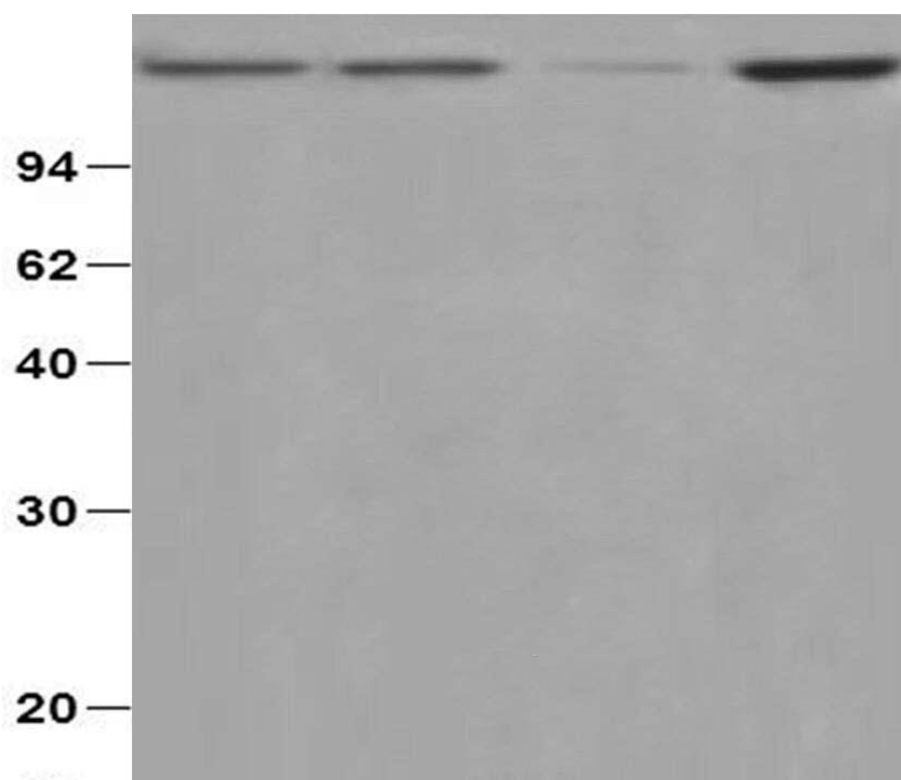

C9

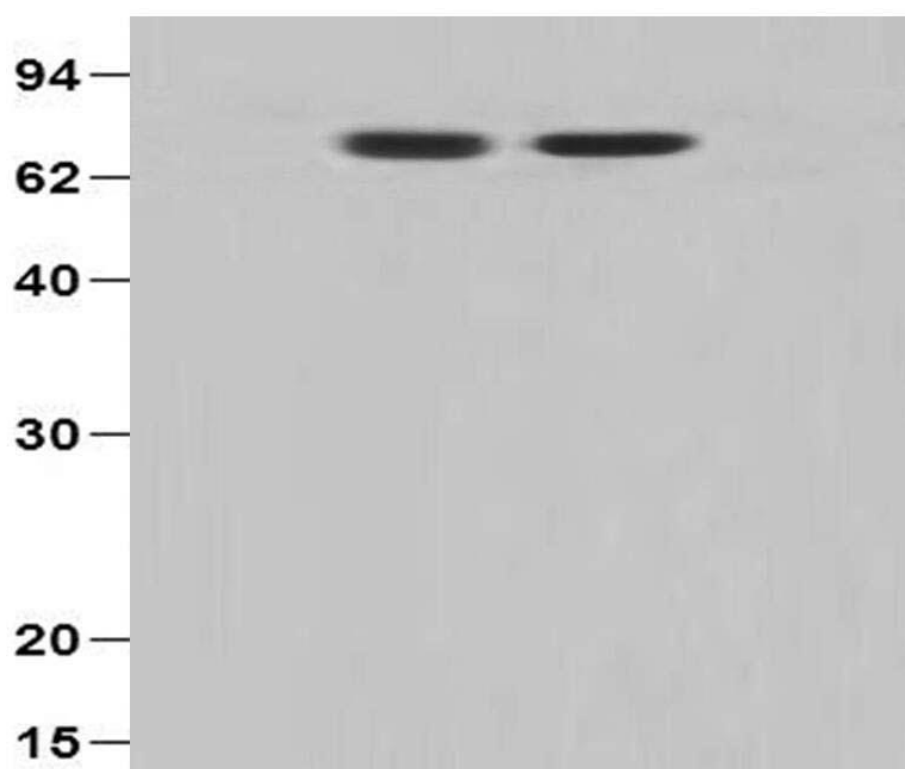

GAPDH1

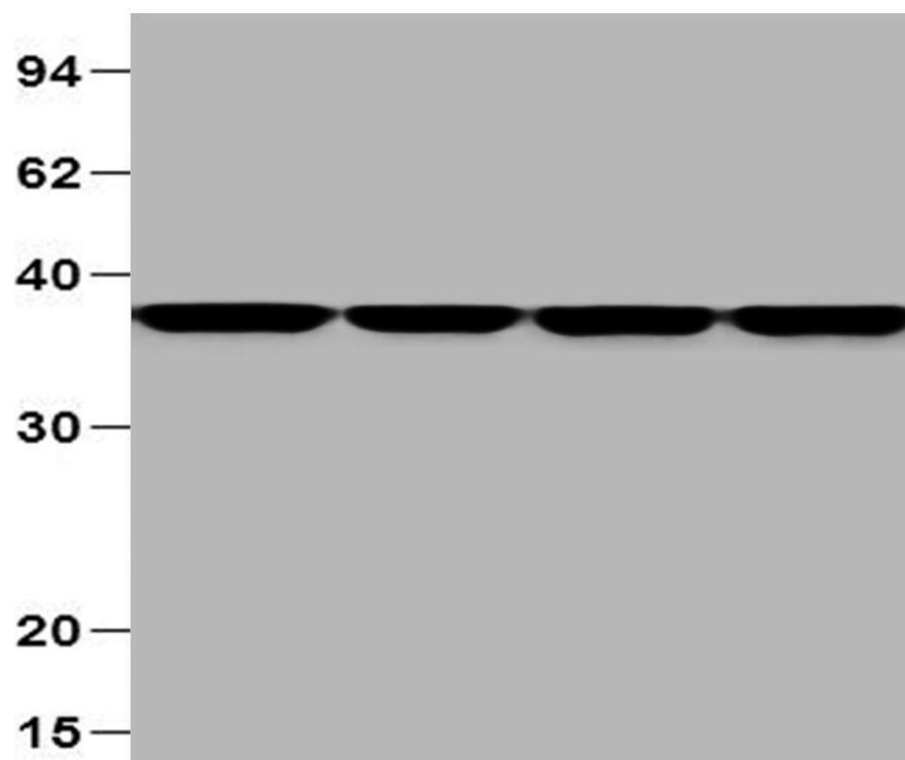

GAPDH2

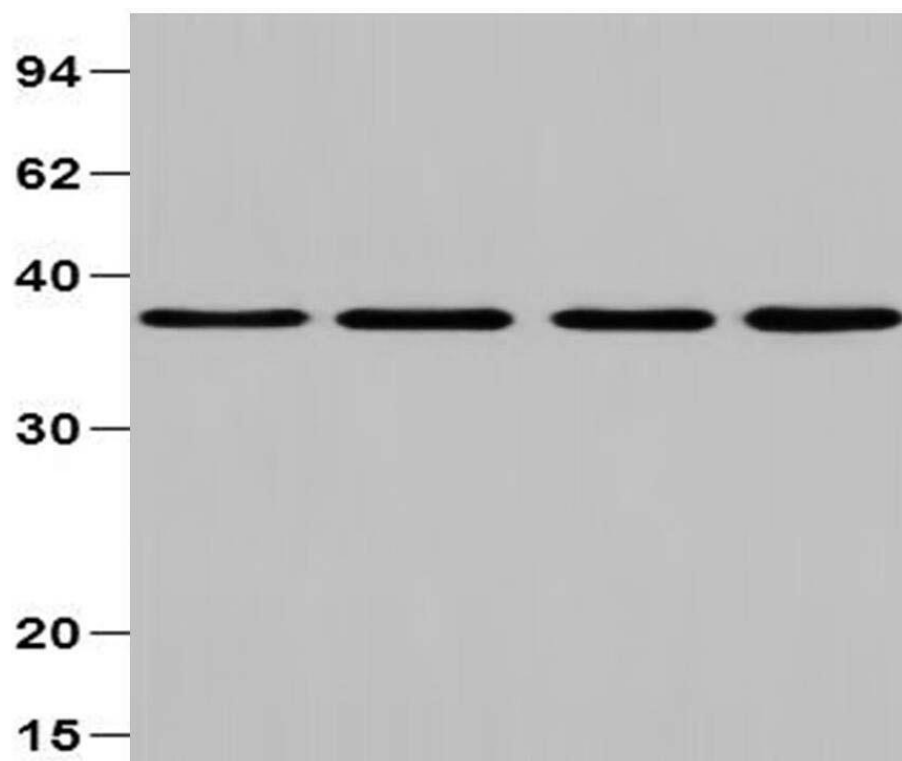

GAPDH3

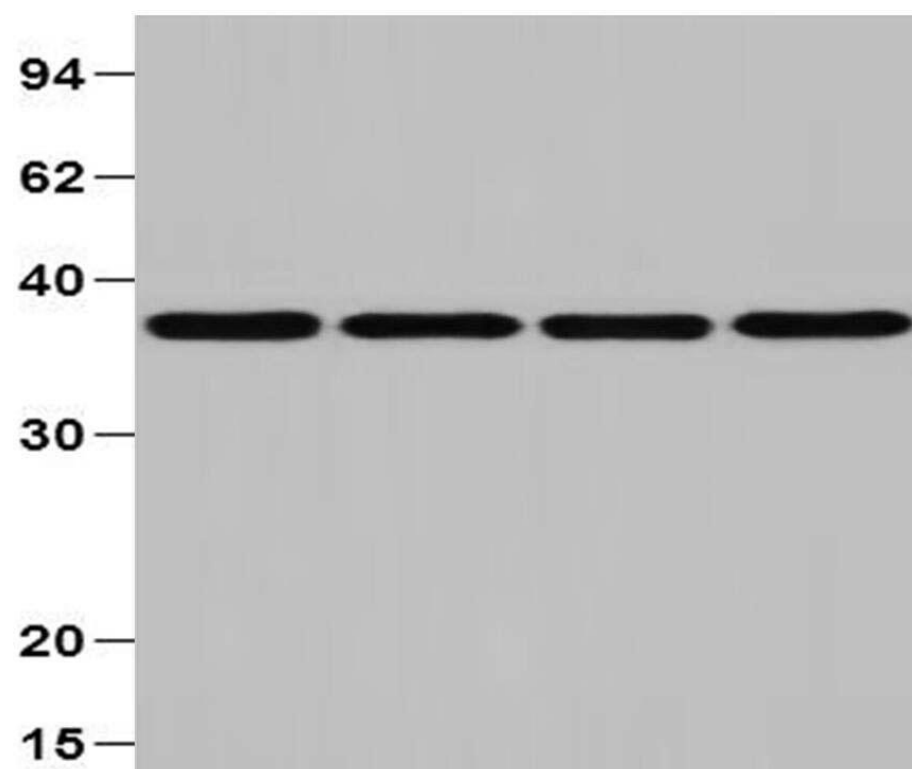

GAPDH4

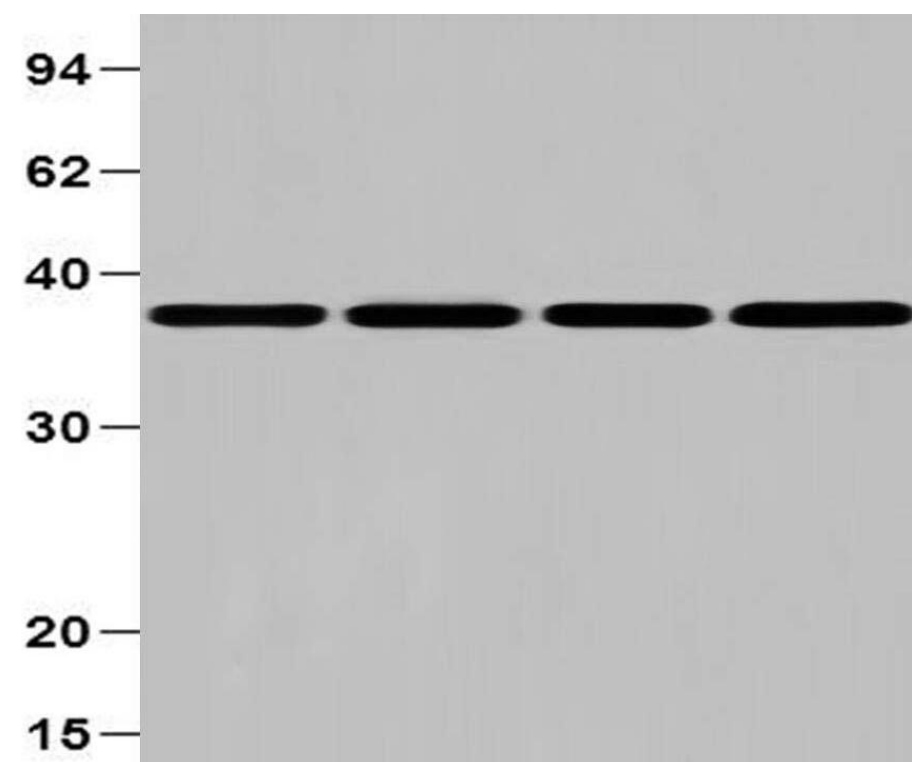

GAPDH5

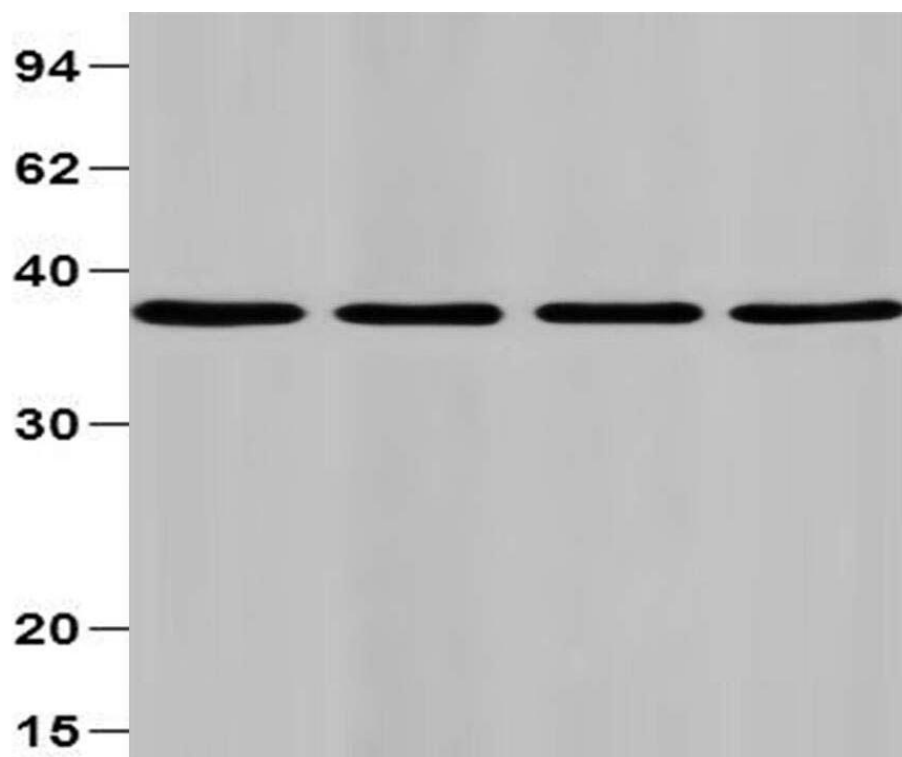

GAPDH6

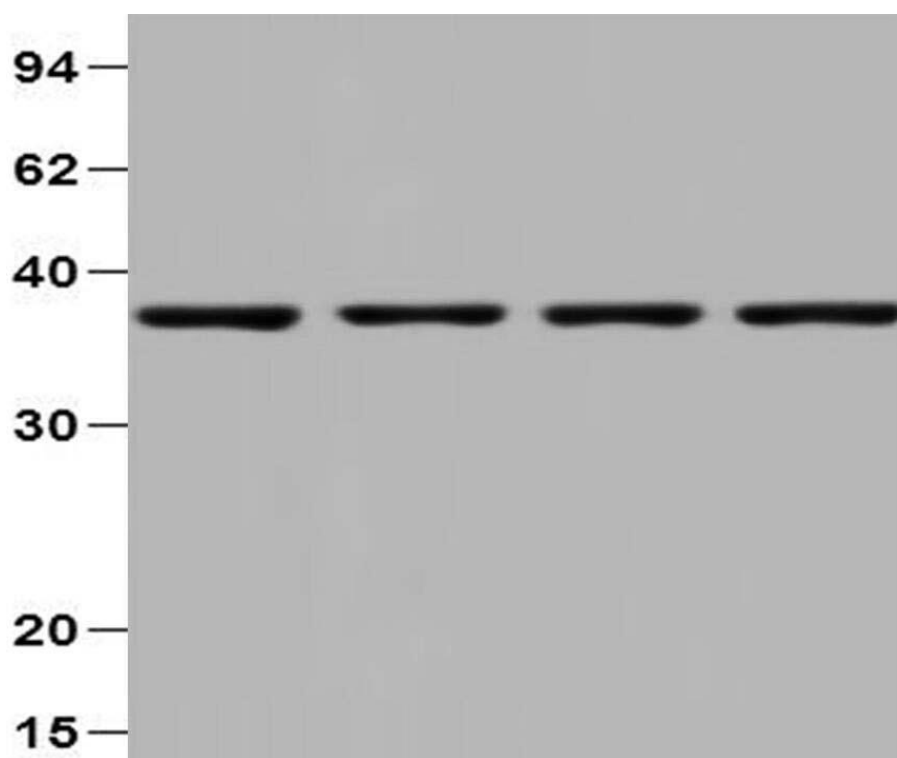

GAPDH7

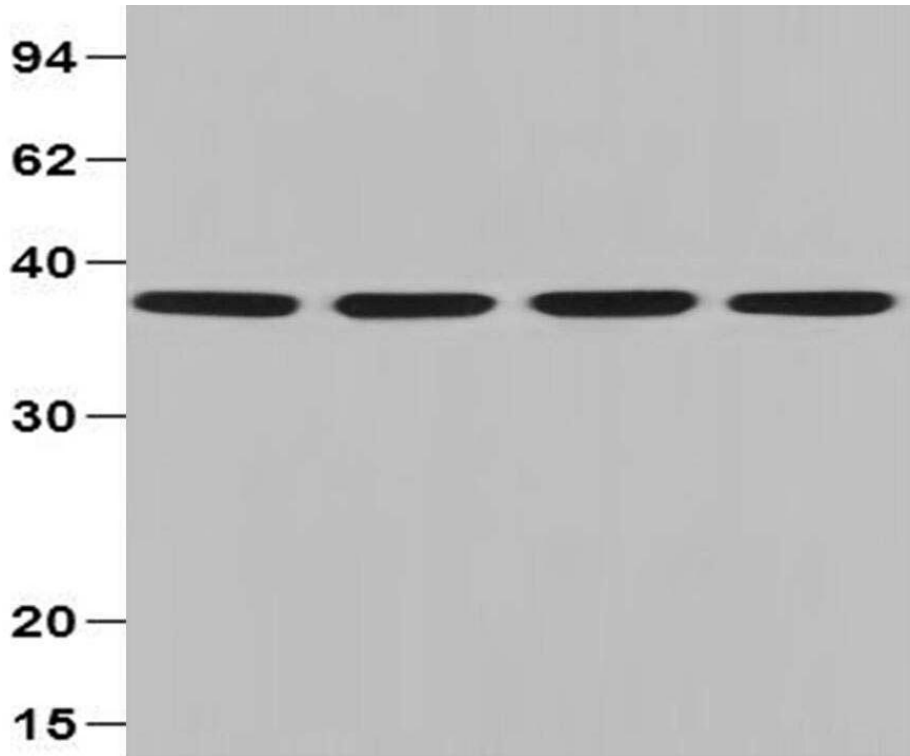

GAPDH8

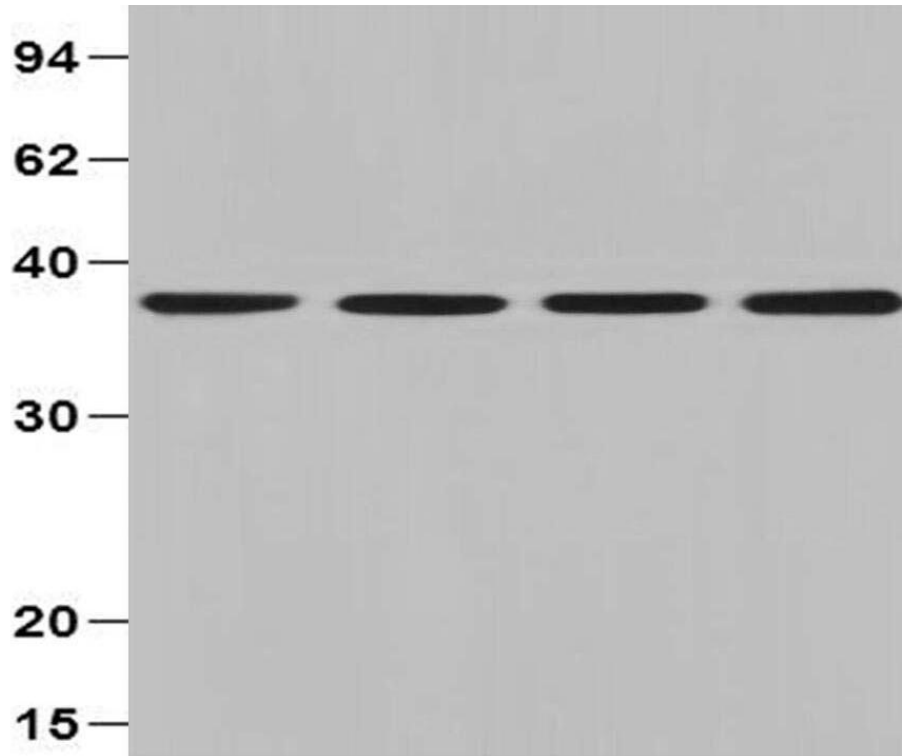

Supplement: Supplementary file 1 [file animals-11-03183-s001.zip › Figure S1 Full figure of WB.pdf]
